# Association between Genetic Variants on Chromosome 15q25 Locus and Several Nicotine Dependence Traits in Polish Population: A Case-Control Study

**DOI:** 10.1155/2015/350348

**Published:** 2015-01-06

**Authors:** Krzysztof Buczkowski, Alicja Sieminska, Katarzyna Linkowska, Slawomir Czachowski, Grzegorz Przybylski, Ewa Jassem, Tomasz Grzybowski

**Affiliations:** ^1^Department of Family Medicine, Nicolaus Copernicus University in Torun, Ludwik Rydygier Collegium Medicum in Bydgoszcz, Sklodowskiej-Curie 9, 85-094 Bydgoszcz, Poland; ^2^Department of Allergology, Chair of Lung Disease, Medical University of Gdansk, Dębinki 7, 80-211 Gdańsk, Poland; ^3^Department of Molecular and Forensic Genetics, Institute of Forensic Medicine, Ludwik Rydygier Collegium Medicum, Nicolaus Copernicus University, Sklodowskiej-Curie 9, 85-094 Bydgoszcz, Poland; ^4^Department of Pulmonary Diseases and Tuberculosis, Nicolaus Copernicus University in Torun, Ludwik Rydygier Collegium Medicum in Bydgoszcz, Seminaryjna 1, 85-326 Bydgoszcz, Poland

## Abstract

Tobacco smoking continues to be a leading cause of disease and mortality. Recent research has confirmed the important role of nicotinic acetylcholine receptor (nAChR) gene cluster on chromosome 15q 24-25 in nicotine dependence and smoking. In this study we tested the association of smoking initiation, age at onset of daily smoking, and heaviness of smoking with five single nucleotide polymorphisms (SNPs) within the CHRNA5-CHRNA3-CHRNB4 cluster. The group of 389 adult subjects of European ancestry from the north of Poland, including 212 ever (140 current and 72 former) and 177 never smokers with mean age 49.26, was genotyped for rs16969868, rs1051730, rs588765, rs6495308, and rs578776 polymorphisms. Distributions of genotypes for rs16969868 and rs1051730 were identical so they were analyzed together. Further analysis revealed the association between rs16969868-1051730 (OR = 2.66; 95% CI: 1.30–5.42) and number of cigarettes smoked per day (CPD) with heaviness of nicotine addiction measured by the Fagerström Test for Nicotine Dependence (FTND) (OR = 2.60; 95% CI: 1.24–5.43). No association between these polymorphisms and other phenotypes was found. Similarly, the association between rs588765, rs6495308, rs578776, and analyzed phenotypes was not confirmed. This study provides strong evidence for the role of the CHRNA5-CHRNA3-CHRNB4 cluster in heaviness of nicotine addiction.

## 1. Introduction

Tobacco smoking is a serious public health concern responsible for approximately 5 million deaths a year [1] and is an important risk factor for 21% of cancers [2] and by the year 2015 is projected to be responsible for 10% of all deaths globally [1].

Evidence from twin studies proved genetic influences on nicotine dependence (ND) [3] and gave impulse to the search for genetic variants that influence ND. This more recent molecular approach indicates that smoking-related phenotypes are highly heritable [4–6].

In previous years genome-wide association studies and some meta-analyses have reported associations between single nucleotide polymorphisms (SNPs) in the CHRNA5-CHRNA3-CHRNB4 nicotinic acetylcholine receptor (nAChR) gene cluster on chromosome 15q 24-25 and some phenotypes describing smoking behavior like ever smoking [7], cigarettes per day (CPD) [6–20], ND defined by the Fagerström Test for Nicotine Dependence (FTND) [4, 5, 7, 11, 14, 21–29], and age at smoking initiation [27, 30, 31].

ND is a leading predictor of smoking continuation [32, 33]. Nicotine, which is a naturally occurring alkaloid in tobacco, affects the body mainly through its influence on nAChRs, for which the natural ligand is acetylcholine [34, 35].

The nicotinic acetylcholine receptor (nAChR), to which nicotine binds, is plausible and biologically relevant candidate for smoking etiology. Neuronal nAChRs are widely distributed throughout the central and peripheral nervous system and brain response to nicotine is caused by their activation. They are ligand-gated ion channels composed of five transmembrane subunit proteins arranged around a central pore. Neuronal nAChR consists of *α* (*α*2–*α*10) and *β* (*β*2–*β*4) subunits [36], each of which is encoded for by a single CHRN gene. The different biological receptor function depends on a kind of subunits combination [37]. Their function might be changed by variation in genes coding for nAChRs, and especially reduced nAChR function may predispose to ND [38, 39].

Association studies can give different results for different populations depending on their structure, genetic heterogeneity, or the selection of participants with a specific disease (e.g., lung cancer or COPD). In this respect, it is worth noting that the majority of association studies performed so far on common variants in nAChR gene cluster employed Western Europeans and Americans of European ancestry, while scarce data has been received so far from populations of Central Europe [40]. Meanwhile, little but visible genetic substructure on the level of autosomal markers exists in Europe, which may affect the results of genetic epidemiological studies, depending on the control samples used [41]. Moreover, associations between common variants and complex phenotypes resulting from genome-wide association studies (GWAS) need to be in principle extensively replicated across different populations [42]. Therefore, we decided to investigate the association between some single nucleotide polymorphisms in the* CHRNA5-CHRNA3-CHRNB4* and a variety of smoking phenotypes in the sample recruited from a general Polish population. To our knowledge, this is the first study focusing on association of common variants in nAChR gene cluster with a variety of smoking phenotypes in a population of Poles.

The aim of this study was to assess the association of smoking initiation (ever versus never smoker), age at onset of daily smoking, and heaviness of smoking (CPD, FTND) with nonsynonymous and intronic SNPs in CHRNA5 (rs588765 and rs16969968, resp.) as well as synonymous, intronic, and 3′UTR SNPs in CHRNA3 (rs1051730, rs6495308, and rs578776, resp.).

## 2. The Study Sample and Measures

### 2.1. Participants

The study sample was collected by the Department of Pulmonology Medical University of Gdansk and Department of Family Medicine, Collegium Medicum in Bydgoszcz, at Nicolaus Copernicus University in Torun. Written consent was obtained from each participant. The study protocol was approved by the institutional ethics committees of Medical University in Gdansk and Collegium Medicum, Nicolaus Copernicus University in Torun. The convenience sample was selected from the consecutive outpatients attending general practices cooperating with the Department of Family Medicine, Nicolaus Copernicus University of Torun, Collegium Medicum in Bydgoszcz, and Academic Clinical Center in Gdansk. With regard to inclusion criteria the participants were 18 years old or older. As for exclusion criteria there were no participants with mental disorders, which might have inhibited the collection of reliable data.

Participants completed a questionnaire referring to sociodemographic data and smoking status. Someone who either had never smoked at all or had never been a daily smoker and had smoked less than 100 cigarettes in his lifetime was considered never smoker [43]. Ever smoker was defined as individual who had smoked at least 100 cigarettes in his lifetime [43]. Individuals, who smoked cigarettes either daily or occasionally, at the time of the survey, were considered to be current smokers [43]. Former smokers were defined as those who had quit smoking at least 1 year before the study.

### 2.2. Phenotypes

Smoking initiation was assessed by the comparison of ever smokers (current or former) with never smokers [17, 43].

Age at onset of daily smoking was evaluated retrospectively among ever smoking participants who were dichotomized into early onset (at age 16 or younger) and late onset (at age 17 or older) [27].

Tobacco consumption was estimated in the group of current smokers with cigarettes smoked per day (CPD). Current smokers were divided into two groups: heavy smokers who smoked 20 or more cigarettes per day and light smokers who smoked less than 20 cigarettes per day [20].

Nicotine dependence was assessed in the group of current smokers with the Fagerström Test for Nicotine Dependence (FTND) score range of 0–10 [21]. Current smokers were divided into low-dependence (0–3 scores) and high-dependence (4–10 scores) according to this scale [6]. FNTD is a widely used test that measures nicotine dependence and in some way predicts difficulties in reducing nicotine level.

### 2.3. Genotype Assessment

Genomic DNA was isolated from blood using* GeneMatrix Bio-Trace DNA Purification Kit* according to the manufacturer's protocols (Eurx, Gdańsk, Poland). DNA quantity was assessed spectrophotometrically with the* Biophotometer* (Eppendorf, Hamburg, Germany). All SNPs were genotyped using commercial* TaqMan SNP Genotyping Assays* (assay IDs: rs16969968: C_26000428_20; rs578776: C_721253_10; rs588765: C_18826_10; rs6495308: C_222570_10; rs1051730: C_9510307_20) on* a ViiA 7 Real-Time PCR System* (Applied Biosystems, Carlsbad, California) following the manufacturer's instructions.

### 2.4. Statistical Analyses

Arlequin v.3.1 software was used to assess deviations of genotype distribution from the Hardy-Weinberg equilibrium.

For analyses, we grouped subjects as carriers and noncarriers, with carriers defined as participants who tested positively for the presence of minor allele, whether homozygous or heterozygous. Logistic regression analysis, using Statistica 7.1 software (StatSoft Inc., USA), was performed to search for associations between genotypes and dichotomous phenotypes, that is, ever smoking, earlier starting of daily smoking (at the age of 16 or earlier), heavier smoking (CPD ≥ 20), and higher dependence (FNTD ≥ 4). The association between phenotypes and genotypes was expressed by odds ratios (ORs) with 95% confidence bounds. Univariate logistic regression analysis was performed to search for other variables potentially associated with the increased risk for examined traits, that is, age and gender. Variables which appeared to be associated with any increased risk for nicotine dependence in the univariate analysis were analyzed by multivariate analysis.

Genotype frequencies were corrected for multiple comparisons using the Bonferroni method, in which the threshold *P* value is obtained by dividing 0.05 by the number of comparisons. In our study *P* value 0.05 was divided by 4; thus, threshold *P* value = 0.0125. We decided to divide it by 4 not 5 because rs16969968 and rs1051730 stay in perfect linkage disequilibrium (LD) block within CHRNA5-A3-B4 in samples of European ancestry (LD decay [*D*′] = 1.0, LD correlation coefficient [*R* ^2^] = 1.0) [15, 17, 22]. So in analyses they are treated interchangeably or together [44].

## 3. Results

Three hundred and eighty-nine adult subjects, including 212 ever smokers (cases) and 177 never smoking controls, were recruited. Among the investigated participants there were 189 women and 200 men; 96 women were ever smokers and 93 were never smokers. The group consisted of 140 current and 72 former smokers, including 65 and 31 female smokers, respectively. The participants were aged from 18 to 87, with the average age being 49.26 (SD = 14.86). All of them were of European ancestry from the north of Poland.

All samples were genotyped successfully. Distributions of genotypes for rs16969868, rs1051730, rs588765, rs6495308, and rs578776 polymorphisms did not deviate to any appreciable extent from expectations predicted by the Hardy-Weinberg equilibrium as determined by Arlequin v.3.1 software (*P* = 0.8172, *P* = 0.8172, *P* = 0.5442, *P* = 0.6455, and *P* = 0.5946, resp.). Distributions of genotypes for rs16969868 and rs1051730 were identical so they were further analyzed together. Genotype frequencies for analyzed SNPs are presented in Table 1.

In order to assess the influence of the investigated polymorphisms on nicotine initiation we evaluated the association between the polymorphisms and the occurrence of smoking phenotype—the comparison of never smokers (*n* = 177) with ever smokers (*n* = 212) [17, 43]. This comparison did not show any significant difference (Table 2) for the analyzed SNPs. Both carriers and noncarriers of minor allele did not differ in this smoking phenotype.

With a view to explore whether the analyzed SNPs have an impact on the age of onset of daily smoking, the group who started smoking was examined, *n* = 212. They were divided into two groups. First group was those who started regular daily smoking at the age of 16 or younger, *n* = 35. The second group was those who started regular daily smoking at the age of 17 or older, *n* = 177. No differences between early and late onset of daily smoking groups were found for all SNPs (Table 2).

Further, the group of current smokers (*n* = 140) was analyzed in terms of the number of cigarettes smoked per day and severity of nicotine dependence. The analysis of the correlation between particular allele and CPD revealed the influence of the polymorphisms rs16969868-1051730 (OR = 2.66; 95% CI: 1.30–5.42) on the daily intake of tobacco: 20 cigarettes or more. In the case of the other polymorphisms, that is, for rs588765 (OR = 0.84; 95% CI: 0.39–1.78), rs6495308 (OR = 0.61; 95% CI: 0.30–1.25), and rs578776 (OR = 0.67; 95% CI: 0.34–1.32), no such correlation was found (Table 2).

In order to assess the influence of the selected polymorphisms on the heaviness of nicotine addiction the group of current smokers was dichotomized into those who obtained 3 points in Fagerström Test (*n* = 45) and those who got 4 or more (*n* = 95). The analysis of the polymorphisms presented in Table 2 revealed a significant influence of the polymorphisms rs16969868-1051730 (OR = 2.60; 95% CI: 1.24–5.43) on the heaviness of nicotine dependence in the group of risk allele carriers. This was not shown for the other polymorphisms, that is, rs588765 (OR = 0.65; 95% CI: 0.28–1.49), rs6495308 (OR = 0.84; 95% CI: 0.39–1.78), and rs578776 (OR = 0.91; 95% CI: 0.44–1.92).

Univariate logistic regression analysis of CPD or FNTD did not show association with age and gender.

## 4. Discussion

Smoking as a substance use disorder is a complex disease with heritability influenced by numerous common polymorphisms, each with only a small to moderate influence on the overall liability to the disorder [45]. Multiple distinct loci at the CHRNA5-CHRNA3-CHRNB4 locus on 15q24-25 appear to affect smoking behavior, and among multiple phenotypes describing nicotine dependence it is genetic factors that are most frequently found responsible for heaviness of smoking [12, 13, 16, 17, 46].

In this study, we have demonstrated and replicated the association between phenotypes describing dependence severity such as CPD and FTND and the polymorphisms rs16969968 and rs1051730.

Tobacco consumption defined by CPD is a strong sign of nicotine dependence and has been the phenotype of choice in the gene association literature. Repeated exposure to inhaled nicotine leads to neuroadaptation; the number of binding sites on the nAChRs in central nervous system increases and this requires the consumption of a larger amount of nicotine [47]. ND requires repeated administration of nicotine [48, 49]. In other words, the greater the nicotine addiction, the more the CPD. In our study carriers of minor allele of rs16969968 and rs1051730 have 2.6 higher odds smoking twenty or more CPD, if compared with noncarriers, which proves the allele's influence on ND severity.

The association between rs16969968 and rs1051730 with heaviness of smoking is also corroborated by the analysis with FNTD, the phenotype often used besides CPD to measure ND severity [21, 50]. FNTD is six-item test and CPD is one of the items. In our study, the chances that carriers of minor allele of rs16969968 and rs1051730 will become high-dependent smokers are more than double (OR 2.60).

Our results presenting the influence of rs16969968 and rs1051730 on ND stay in agreement with previous evidence from extensive studies [12, 13, 16, 17, 46]. Some studies analyzed ND severity measured by means of CPD [6, 12, 13, 17, 46] and others by FNTD [51], and in others both phenotypes were analyzed [20]. They indicate that the polymorphisms rs16969968 and rs1051730 which remain in a strong LD are related to heaviness of smoking and minor allele may be responsible for difficulty in smoking cessation.

This phenomenon for rs16969968 is well explained by in vitro tests [38, 52], which showed that nicotine receptors which contain CHRNA5, with aspartic acid (D398), respond better to the influence of agonist than asparagine-containing receptors (N398). CHRNA5 containing asparagine changes the receptor's function, leading to a prolonged desensitization and reduced transmission of calcium ions [39, 52]. The decreased function of nicotine receptors results in the necessity of the increased nicotine intake and, as an effect, the development of ND [22, 26, 40, 46, 53].

In the case of the other polymorphisms rs588765, rs6495308, and rs578776 we did not find any statistically significant association between ND severity and the other analyzed phenotypes.

The existing literature confirms this association for the polymorphism rs6495308 in the meta-analysis conducted by Liu et al. [12] and in the study by Bousman et al., in which heavy smokers, that is, participants smoking 25 or more CPD, had approximately 20% greater odds of being minor allele carriers [19]. The lack of association between this polymorphism and CPD in our study may result from the applied cutoff of 20 or more, so that the groups of smokers compared were slightly different. We adopted 20 CPD as a cutoff point because in most research studies heavy smokers are individuals smoking 20 cigarettes or more. Besides, the study by Bousman et al. [19] was conducted on European-American smokers, while ours was conducted only on individuals of European ancestry. It follows from autosomal DNA studies employing thousands of SNPs that American-European populations reflect a complex pattern of genetic structure and admixture which affect association studies [54]. A similar association between rs6495308 and CPD in non-European population, specifically in Korean male population, was demonstrated by Li et al. [55].

On the other hand, the association between rs578776 and smoking dependence was shown in the study of the US and Australian Europeans by Saccone et al., but in the case of this polymorphism the higher level of ND expressed in FNTD ≥ 4 occurred more often in the participants with major allele (OR 1.31), rather than in those with minor allele (OR 0.75) [51]. It is possible to contend then that minor allele has a protective function against the development of ND. Similar observations, but with reference to CPD, were presented in a population study by Hällfors et al. [56]. In our study this association was not found, which may possibly be explained by the size of the sample, which was too small to observe this association or existing genetic heterogeneity between different populations of European ancestry.

The role of rs588765 in ND was unconfirmed in our study and its association with CPD in the literature is unclear. The association between CPD and rs588765 was observed in the group smoking more than 30 CPD in the study by Saccone et al., but further research and meta-analyses did not replicate this [12, 13, 17, 56].

No SNPs in our study were significantly associated with ever versus never smoking. This seems to be related to the fact that the division into ever and never smokers is too general. In the group of never smokers there were both individuals who had smoked less than 100 cigarettes in their lifetime and those who had never smoked even one puff. Likewise, the group of ever smokers comprised both individuals who smoked for a short period of time but smoked at least 100 cigarettes and those who smoked for many years. It is worth drawing attention to the fact that it is usually environmental and personality factors that affect smoking initiation as well as first cigarettes smoking [57]. Biological factors are likely to be more important when it comes to the persistence of ND, rather than smoking initiation or even smoking cessation [19, 51]. Therefore, the results of our study are concordant with previous studies, which did not corroborate the association between the analyzed SNPs and smoking initiation either, yet they demonstrated the association existing for ND severity [17].

Adolescence is a crucial period of vulnerability for the development of substance use disorders [58]. People who begin daily smoking at an early age are at greater risk of long-term nicotine addiction. The early onset of smoking is associated with greater consumption of cigarettes in adulthood [59], a relative inability to quit smoking [60], and a more severe form of ND [61].

The results of this study have not corroborated the association between the analyzed SNPs and starting regular smoking at the age of 16 or younger compared with starting regular smoking at the age of 17 or older. Previous results have been inconsistent in finding support in the CHRNA5-CHRNA3-CHRNB4 variation underlying ND in individuals with early versus late nicotine exposure.

Grucza et al. found that SNP rs16969868 in CHRNA5 was associated with later onset of daily smoking [30]. This contrasts with the findings of Weiss and colleagues who showed that this polymorphism was more important among early onset smokers [27]. This discrepancy in the results can possibly be explained by the influence of environmental factors on the age at first use or age at onset of regular use. Nevertheless, it is possible that the study conducted on large population samples can significantly rule out differences in sampling as well as in random fluctuation.

## 5. Limitation of the Study

The main limitation of our study was a small sample size as the sample size in thousands of participants is likely to be more appropriate to reveal small genetic effects, which are likely for single loci and complex smoking behavior. However, several genetic association studies on smoking were conducted using comparable samples, which consisted of several hundreds of participants, and positive associations were reported [62, 63]. It seems that studies on smaller populations may be sufficient for obtaining reliable results in the case of a precisely defined phenotype, for example, CPD, and for robust association of this phenotype with genotype. On the other hand, smaller samples have suboptimal power to detect significant genotypic effects for smoking related behaviors when the relation between genotype and smoking is not strong or if the phenotype does not describe smoking behavior sufficiently.

Despite this main limitation, these findings significantly contribute to our current knowledge of nicotinic receptor gene variation and smoking behavior and are comparable with the findings on larger populations.

The second limitation is participants' self-reported smoking status and other smoking related behaviors. A potential bias can occur due to a socially desirable response, which is hiding the fact of smoking tobacco. Certainly the confirmation of declared information by objective tests, such as measurement of carbon monoxide or cotinine level, would best verify the data but on the other hand previous research has shown that self-reported data about smoking are generally reliable [64].

## 6. Conclusion

Since much effort should be invested in replicating associations between common genetic variants and complex phenotypes across populations, we presented here the results of association of common variants in the nAChR gene cluster and a variety of smoking phenotypes in a Polish population. Our findings show that rs16969968 and rs1051730 single nucleotide polymorphisms in the CHRNA5-CHRNA3-CHRNB4 nicotinic acetylcholine receptor gene cluster on chromosome 15q 24-25 influence nicotine dependence assessed with the number of cigarettes smoked per day and the Fagerström Test for Nicotine Dependence. Since no traces of hidden population substructure were found in Poles on the level of autosomal markers, we consider our results reliable and representative of the general population of Poland. Therefore, associations replicated in our study might help in the future in adopting a more tailored approach to treat addicted Polish patients, while taking account of their genetic predisposition for smoking.

## Figures and Tables

**Table 1:** Genotype frequency distribution.

Polymorphism	Genotype	Number of subjects *N* (%)
rs16969868^a^	AA	42 (10.80)
	AG	169 (43.44)
	GG	178 (45.76)

rs1051730^a^	AA	42 (10.80)
	AG	169 (43.44)
	GG	178 (45.76)

rs588765^b^	TT	80 (20.57)
	TC	200 (51.41)
	CC	109 (28.02)

rs6495308^c^	CC	15 (3.86)
	CT	133 (34.19)
	TT	241 (61.95)

rs578776^a^	AA	28 (7.20)
	AG	144 (37.02)
	GG	217 (55.78)

^a^Risk (minor) allele A.

^b^Risk (minor) allele T.

^c^Risk (minor) allele C.

**Table 2:** Association between genotypes and smoking-related phenotypes.

SNP	Smoking initiation *N* = 389			Age at onset of daily smoking *N* = 212			CPD *N* = 140			FNTD *N* = 140		
	Never	Ever	OR (95% CI)	≤16 years	>16 years	OR (95% CI)	<20	≥20	OR (95% CI)	<4	≥4	OR (95% CI)
16969868^a^-1051730^a^												
AA + AG	88	123	1.39 (0.93–2.02)	20	103	1.04 (0.49–2.18)	35	52	2.66 (1.30–5.42)^*^	21	66	2.60 (1.24–5.43)^**^

588765^b^												
TT + TC	129	151	0.92 (0.59–1.44)	27	123	0.69 (0.29–1.63)	51	50	0.84 (0.39–1.78)	35	66	0.65 (0.28–1.49)

6495308^c^												
CC + CT	75	73	0.71 (0.47–1.07)	12	61	1.01 (0.46–2.23)	27	20	0.61 (0.30–1.25)	16	30	0.84 (0.39–1.78)

578776^a^												
AA + AG	85	87	0.68 (0.45–1.05)	15	72	1.09 (0.52–2.29)	32	26	0.67 (0.34–1.32)	19	38	0.91 (0.44–1.92)

FNTD: Fagerström Test for Nicotine Dependence; CPD: cigarettes per day.

^*^ *P* = 0.007.

^**^ *P* = 0.01.

Genotype frequencies were corrected for multiple comparisons using the Bonferroni method. We analyzed rs16969968 and rs1051730 together because they stay in perfect linkage disequilibrium block. So the threshold *P* value was obtained by dividing 0.05 by 4; thus, threshold *P* value = 0.0125.

^a^Risk (minor) allele A.

^b^Risk (minor) allele T.

^c^Risk (minor) allele C.
